# Prospective uncontrolled clinical study shows rapid and long lasting relief of heartburn and acid related gastric discomfort with Refluthin

**DOI:** 10.1038/s41598-025-98558-z

**Published:** 2025-05-07

**Authors:** Ahmed Madisch, Fabio Pace, Daniel Menzel, Petra Funk, Berenike Stracke, Christiane Schön, Joachim Labenz

**Affiliations:** 1 Gastroenterology Center Bethanien, Im Prüfling 21-25, 60389 Frankfurt am Main, Germany; 2 GI Unit, Bolognini Hospital, Via Paderno 21, 24068 Seriate, Bergamo Italy; 3 Nutritional CRO, BioTeSys GmbH, Schelztorstrasse 54-56, 73728 Esslingen, Germany; 4 Research and Development, Dr. Willmar Schwabe GmbH & Co. KG, Willmar-Schwabe-Straße 4, 76227 Karlsruhe, Germany; 5 Global Medical Affairs, Dr. Willmar Schwabe GmbH & Co. KG, Willmar-Schwabe-Straße 4, 76227 Karlsruhe, Germany; 6 Medical Center, Flughafenstraße 2/2a, 57299 Burbach, Germany

**Keywords:** *Opuntia ficus-indica* extract, Heartburn, Acid regurgitation, Gastro-esophageal reflux, Symptom relief, Refluthin, Gastrointestinal diseases, Medical research

## Abstract

Heartburn and acid regurgitation are main symptoms of gastro-esophageal reflux, a widespread complaint with a significant impact on quality of life (QoL). *Refluthin® for Heartburn* chewable tablets (Refluthin) are a symptomatic treatment option containing a combination of an antacid (CaCO_3_, MgCO_3_) and a polysaccharide-rich extract with mucoprotective substances from *Opuntia ficus-indica* cladodes. To investigate performance and safety of Refluthin in the rapid and long lasting relief of heartburn and acid related gastric discomfort under practical use conditions, a prospective, clinical, uncontrolled, open-label study was conducted. Adults with symptomatic heartburn, acid regurgitation, and/or recurrent acid related gastric discomfort took one tablet Refluthin up to four times/day as needed for up to 4 weeks. Endpoints were both time to onset and duration of symptom relief; reflux symptom intensity/frequency; global assessments (QoL, performance, satisfaction, usability, tolerability); and safety. 81/100 participants (81%) responded with a first symptom relief within ≤ 20 min in ≥ 50% of the individual applications. Long lasting effects of > 120 min were reported by 83/100 (83%) participants. Significant reductions in heartburn event frequency and intensity were seen within the 4 weeks of intermittent use (*p* < 0.0001, respectively). Global assessment results and safety-relevant findings were also favorable. Results thus demonstrated a distinct rapid and long lasting symptom relief after intake of Refluthin, with a safe and easy use. The significant reductions in frequency and intensity of heartburn events over time indicate sustained effects under treatment. These long-term effects might be explainable by soothing and protection of the irritated mucous membrane by Refluthin.

## Introduction

Heartburn and regurgitation are typical symptoms of gastro-esophageal reflux (GER). Overall, 10–20% of the Western population are affected by at least weekly heartburn and/or acid regurgitation^1,2^. For Germany, the overall prevalence of moderate and severe reflux symptoms in adults was shown to be 14% and 4%, respectively^3^. Patients exhibit a diminished health-related quality of life (QoL) and activity impairment, which leads to high healthcare and economic costs due to a reduced patient productivity in both work and daily activities, even in cases of moderate or mild reflux symptoms^4^.

The typical GER symptoms of heartburn and acid regurgitation arise because of a retrograde flow of gastric contents into the esophagus, associated with disruption of mucosal barrier integrity and sensitization of nociceptive chemoreceptors^5,6^. Gastric juice as a caustic mixture of acid, bile, and digestive enzymes can injure epithelial layers if it overcomes appropriate protective measures that can buffer the acid and protect the mucosae^5^. With repeated exposure, secondary inflammatory responses of the epithelium contribute to sensitization by impairment of epithelial cell tight junction expression or by modulation of the activity of transient receptor potential channels on nociceptors^5^.

Non-pharmacological treatment of GER-related heartburn and acid regurgitation involve lifestyle modifications ranging from bed head elevation and quitting smoking to change in eating habits and weight reduction in overweight/obese patients^7–9^. First-line pharmacological treatment is acid suppression, often achieved through suppression of acid production by proton pump inhibitors (PPI)^10^. The German S2k-Guideline recommends that alternative treatment options (e.g., alginates or antacids) should also be considered or even preferred if they are sufficiently effective^8^. According to this guideline, treatment with e.g. antacids or alginates is appropriate for typical reflux symptoms without alarm signs if this provides sufficient symptom control from the patient’s perspective. The World Gastroenterology Organisation recommended PPI only in patients with regular and/or severe symptoms^9^. Moreover, gastric juice can remain acidic in patients given high doses of PPI, and this so-called weakly acidic reflux is still involved in the generation of symptoms such as regurgitation and heartburn^5,11^.

In patients with mild symptoms, self-medication with antacids is common and popular^12^. Antacids are mineral salts containing pH-increasing/neutralizing anions accompanied by aluminum, calcium, magnesium, or sodium cations. Unlike PPI, antacids do not suppress the production of hydrochloric acid by the gastric parietal cells, but directly neutralize acidity of the gastric reflux^12^. Antacid therapy was demonstrated to lead to a rapid neutralization of the backwashing acidity and short-term symptom improvement^10,13^.

Another possible treatment target is mucosal barrier protection, as sensory mechanisms determine the relationship between reflux exposure and symptom generation^5^. In this context, therapeutic preparations containing constituents of cladodes (i.e., stems) of *Opuntia ficus-indica* (L.) Mill. are considered promising. *Opuntia ficus-indica* (prickly pear, nopal cactus) is a plant belonging to the Cactaceae family and usually growing in dry climatic zones especially in the Mediterranean and Central America regions^14^. In Sicily traditional medicine, cladodes of *Opuntia ficus-indica* (L.) Mill. are used for gastric ulcer treatment. The traditional use is confirmed by scientific studies demonstrating that polysaccharides from Opuntia are mucoprotective due to their capability to act as a protective layer on mucosal surfaces, accelerating the re-epithelization of dermal wounds^14–16^. For preparations based on Opuntia cladodes, protective activity was demonstrated in animal models of experimentally induced gastritis^15,17–19^. Moreover, fractionation studies showed that the protective effect was mediated by the mucilage fraction containing high molecular weight polysaccharides^17,19^. The clinical relevance of the observed activities was confirmed in two double-blind, randomized, controlled studies demonstrating efficacy of products containing a polysaccharide-enriched Opuntia extract in the treatment of patients suffering from GER disease (GERD) and gastrointestinal discomfort, respectively^20,21^.

Against this background and anticipating a dual mode of action, an antacid/Opuntia extract combination appears to be a promising treatment approach. A corresponding combination of an antacid (calcium carbonate, CaCO_3_; magnesium carbonate, MgCO_3_) and a highly concentrated extract with plant-based protective substances from *Opuntia ficus-indica* (L.) Mill. (OPUNXIA® 70, Bionap SRL; Italy) is contained in *Refluthin® for Heartburn* chewable tablets (Refluthin)^22,23^. Intended use of Refluthin as defined in the instructions for use (IFU) is the rapid and long lasting relief of heartburn and recurrent acid related gastric discomfort. Non-clinical study results show that the polysaccharide-rich extract containing protective substances from *Opuntia ficus-indica* cladodes, which is included in Refluthin, is mucoprotective and does not decrease the acid-buffering effect of the antacid^23,24^.

While clinical trials showing the beneficial effect of products containing CaCO_3_ and MgCO_3_ have already been published^10,25^, clinical data for a combination product involving *Opuntia ficus-indica* extract OPUNXIA® 70 as provided by Refluthin are still lacking. Therefore, a post-market clinical follow-up (PMCF) study was carried out to investigate the performance and safety of Refluthin in the improvement of heartburn, acid regurgitation, and/or recurrent acid related gastric discomfort under conditions of practical use.

## Materials and methods

### Study design

The prospective, single-arm, uncontrolled, open-label study was performed at the study site of BioTeSys GmbH (Esslingen, Germany) between July 2022 and March 2023. It was carried out to investigate the performance and safety of Refluthin in the improvement of heartburn, acid regurgitation, and/or recurrent acid related gastric discomfort in adults during 4 weeks under conditions of normal use, i.e., adhering to the IFU. The study was not designed to test hypotheses but focused on the use under market conditions and on symptom control perceived by the patients. Therefore, a non-confirmatory study design without a placebo arm was chosen.

### Legal requirements and ethics

The study was conducted with the approval of the Ethical Committee (Landesärztekammer Baden-Württemberg, May 18, 2022; reference no. F-2022-038). It was prospectively registered at the German Clinical Trials Register (DRKS, identifier DRKS00029344) and carried out in accordance with the principles of the Declaration of Helsinki, the Professional Code for Physicians in Baden-Württemberg, and in orientation to the International Organisation for Standardization (ISO) 14155:2020 (Clinical investigation of medical devices for human subjects).

### Participants

Male or female subjects aged ≥ 18 years and suffering from symptomatic heartburn, acid regurgitation, and/or recurrent acid related gastric discomfort with at least 1 heartburn event per week were included. Further inclusion criteria were an indication for use of Refluthin according to the IFU and the investigator’s judgement, and the participant’s willingness not to use any other medication or medical device for treatment of heartburn and acid reflux symptoms during the study. Exclusion criteria were: a history of GERD requiring or having previously required treatment with prescription medications, surgery or endoscopic therapy; a history of heartburn or acid reflux symptoms caused by prescription medication, Barrett’s esophagus, hiatal hernia, esophagitis, or cancer; hypersensitivity or allergy to any of the product ingredients; hereditary fructose intolerance; renal disorders such as kidney stones or renal dysfunction; known high blood or urine calcium concentrations; known low blood phosphate level; intake of thiazide diuretics (indicated for high blood pressure or cardiac insufficiency); inability to follow study procedures (e.g., due to language barrier or mental/psychological disorders); participation in any clinical investigation in the last 30 days prior to the study. Pregnancy was no exclusion criterion. Study participants were free to withdraw from the study at any time and with no consequences.

### Endpoints

Endpoints for performance assessment were time to onset of symptom relief (first relief and acceptable symptom relief, respectively; assessed by study participant; event diary; per event); duration of effect (assessed by study participant; event diary; per event); number of events and days with an event (assessed by study participant; event diary); number of tablets taken during the study and intake per event (assessed by study participant; event diary); intensity of heartburn symptoms (assessed by study participant; event diary); symptom frequency and intensity (assessed by study participant; validated Reflux Symptom Questionnaire 7-day recall (RESQ-7)^26^; day 7 (D7), 14 (D14), 21 (D21), 28 (D28)); global performance overall and based on the effect on the 4 single heartburn symptoms, chest pain, burning sensation, regurgitation, and acid taste in the mouth, global satisfaction, and global QoL (each assessed by study participant; global scaled evaluation; D28); as well as willingness of the study participant to recommend the product to a friend (assessed by study participant; global scaled evaluation; D28). Due to the non-confirmative design, there was no formal primary endpoint. Adverse events (AEs), adverse device effects (ADEs), and device deficiencies were documented for safety assessment. Further safety endpoints were global tolerability (participant’s and investigator’s assessment, respectively; global scaled evaluation; D28) and the global assessment of application/user-friendliness (participant’s assessment; global scaled evaluation; D28).

### Medical device under investigation

Refluthin is a medical device for the rapid and long lasting relief of heartburn and recurrent acid related gastric discomfort and intended for on-demand, short term use in adults and children from 12 years on. One chewable tablet of Refluthin contains 450 mg CaCO_3_, 50 mg MgCO_3_, and 75 mg *Opuntia ficus-indica* extract as principal ingredients to achieve the intended purpose. The MoA is classified as antacid since the principal ingredients neutralize acidic stomach content. In Germany, Refluthin is CE-marked under the Medical Device Directive since 2021. According to the IFU, the recommended maximum dose of four tablets/day should not be exceeded. If ingested daily, Refluthin should not be used for more than 14 consecutive days to avoid high calcium intake. In case of intermittent use, Refluthin should not be used longer than 30 days.

### Study procedures

Subjects recruited via the database of the contract research organization, flyers, and media advertisements who met the pre-screening criteria checked by telephone were screened at the study center (day 0 (D0), visit 1). Written informed consent was obtained and subjects meeting all inclusion and none of the exclusion criteria were enrolled. Physical examination was performed and relevant personal and medical data (e.g., medical history, concomitant treatment, and date of a previously made heartburn diagnosis, if applicable) were recorded. Regular concomitant treatment was allowed, except for products conflicting with Refluthin use (e.g., other products used for heartburn symptoms, products with high calcium content).

Symptoms occurring prior to the study were assessed (Short-GER questionnaire; 5-point scale: 0 = “never” to 4 = “daily”; range 0–20 points). Participants specified any previous therapies or surgical interventions related to their GER symptoms and rated their overall QoL with respect to the past month (“excellent”, “very good”, “good”, “fair”, or “poor”). They also assessed symptom frequency and intensity via the RESQ-7, a 13-items patient-reported outcome instrument developed in line with US Food and Drug Administration guidelines for the assessment of GERD symptoms over the past 7 days^26^. Intensity of each symptom was rated on a six-point scale (0 = “did not have” to 5 = “severe”). Frequency was scored as the number of days each symptom was experienced in the previous 7 days (have not had; 1 day; 2 days; 3–4 days; 5–6 days; daily). Participants were handed out the RESQ-7 for D7, D14, and D21 and instructed to complete them at home at the respective day. The German version of the RESQ-7 was provided and permission for its use was granted by AstraZeneca R&D, HEOR, Mölndal, Sweden.

Study participants then received Refluthin (2 packages with 48 tablets each and 1 package with 16 tablets) in their preferred flavor (mint or fruit; in cases of peppermint allergy, fruit flavored tablets were handed out). According to the IFU, participants were instructed to take one Refluthin tablet up to 4 times daily as needed, over a 4-week study period and starting immediately after enrollment. Moreover, the participants were handed out event diaries to record the following information each time they used Refluthin: number of tablets taken, intensity of initial heartburn symptoms (11-point scale; 0 = “no symptoms” to 10 = “very strong symptoms”), time to onset of symptom relief (9-point scale; “less than 5 min after ingestion” to “never”), time to acceptable symptom relief (9-point scale; “less than 5 min after ingestion” to “never”), severity of symptoms after the onset of the acceptable symptom relief (11-point scale; 0 = “no symptoms” to 10 = “very strong symptoms”), and duration of effect (4-point scale; “less than 30 min” to “more than 120 min”).

Visit 2 was scheduled as a remote phone interview on D14 ± 2. Study participants were reminded to complete the RESQ-7 for the same day, and any AE, ADE, and device deficiency having occurred during D0 to D14 was documented. Concomitant treatment and the number of days with Refluthin use within the past two weeks were recorded. In accordance with the IFU, the clinical investigation was ended for participants who had taken Refluthin daily through 14 days prior to visit 2, and the final visit was scheduled promptly. All other participants continued the study. They were asked to fill in the RESQ-7 on D21 and to report to the investigator in case they had taken Refluthin uninterruptedly for 14 days during the continued treatment period.

The final visit (visit 3, at study center) was scheduled on D28 ± 2. Study participants completed the RESQ-7 and the global assessments regarding performance (“very good” to “no effect”), satisfaction (“very satisfied” to “unsatisfied”), and recommendation (“would strongly recommend” to “would not recommend”) at the study site. They were also asked to globally assess application/user-friendliness, i.e. if Refluthin was easy to use (“easy-to-use”; “not easy-to-use”; “indifferent/no decision”), easy to be integrated into daily routine (“easy to be integrated”; “not easy to be integrated”; “indifferent/no decision”), and easy to understand according to the IFU (“easy to understand (IFU)”; “not easy to understand (IFU)”; “indifferent/no decision”). Additionally, they rated their overall QoL over the past month (“excellent” to “poor”) and were asked if regularly using Refluthin did have an impact on their overall rating of QoL (“yes”; “no”). Global assessment of tolerability was performed by both participants and investigator (“poor” to “very good”). In addition, concomitant treatment was recorded and any AEs, ADEs, or device deficiencies having occurred since the last visit was assessed by the investigator. The event diary and the completed RESQ-7 forms were returned and checked for completeness.

### Statistical analysis

Due to the non-confirmatory nature of this study, which was designed to evaluate the performance and safety of Refluthin under practical conditions, there was no defined primary outcome measure. Nevertheless, we felt it was important to base our sample size calculation on solid data. In line with the relevant German S2k guideline^27^, the study focused on symptom control as well as global assessments by patients. Neither for these parameters nor for a study population such as ours could we derive any statement about the clinical relevance from the literature. Sample size calculation was therefore based on the RESQ-7 in a GERD population, which was the most suitable parameter found. For the calculation, data and considerations from earlier reported trials^28,29^ were taken into account: Villamil Morales and colleagues^28^ used the assumption that the intervention would produce a difference of at least 10% in the Reflux Disease Questionnaire (RDQ), on which the development of the RESQ-7 was based^30^. The authors calculated the effect size by taking into account the mean and standard deviation for the RDQ of 3.3 ± 1.0 points previously found in a Spanish population with symptomatic GERD^29^. By applying these considerations for the present study, we assumed a clinically important difference of 10% (0.5 points in RESQ-7 (range 0–5)), which resulted in a sample size of N = 100. Moreover, applying a 10% reduction in symptom intensity, a standardized effect size of 0.33 points was derived for the present study. Assuming a drop-out rate of 25%, the power to detect a minimum standardized effect difference of 0.33 within a one-group comparison, a significance level α = 0.05, and applying a two-sided paired t-test, was 80%. All analyses were descriptive, and *p* values should be interpreted accordingly.

The intention-to-treat (ITT) population/full analysis set (FAS) included all participants who had used Refluthin at least once and for whom at least one assessment under treatment was available. The safety population for safety analysis included all participants applying Refluthin at least once. To avoid any bias due to the different time frames of Refluthin use, all endpoints assessed or calculated as averages on a weekly basis are presented separately for patients with intermittent use (IU) and those with consecutive use (in the following referred to as daily use (DU), which here also means consecutive use for 14 days after a preceding period of IU). Responder rates are presented for the total study population. Global assessments are presented for IU, DU, and the total population.

A descriptive evaluation of baseline characteristics was performed for demographic data (sex, age, BMI, alcohol and coffee consumption), Short-GER questionnaire, concomitant treatment, medical history, and anamnesis concerning heartburn symptoms, frequency of heartburn events prior to the study, heartburn triggers, and heartburn-related medical treatment prior to the study. The number of events, tablets taken, and days with an event, as well as the use of Refluthin per event were derived from the event diaries. For intensity of initial heartburn symptoms, intensity of symptoms after the onset of the acceptable symptom relief, time to onset of symptom relief (first/acceptable), and duration of the effect, an individual mean value was calculated from all events over the whole treatment period as well as for week 1 (W1), week 2 (W2), week 3 (W3), and week 4 (W4). For participants for whom the study was ended after 14 days of daily Refluthin use, single events affiliated to W3 were considered in W2 assessments. The two-sided paired t-test was applied to compare the intensity of symptoms before and after the intake of Refluthin for the whole observation period as well as per week. Furthermore, descriptive statistics were evaluated on group level over the whole treatment period as well as per week.

Additionally, responder analyses were calculated event-based for the time to onset of first symptom relief and acceptable relief, as well as the duration of symptom relief. Based on different cut-off values, a responder was defined if the appropriate onset or duration of relief was reported within the specified cut-off category in ≥ 50% of reported events.

RESQ-7 mean overall symptoms domain scores (OSDS) for intensity and frequency were calculated descriptively for each time point. For calculation of frequency, the response options ‘3–4 days’, ‘5–6 days’, and ‘daily’ were recoded into 3.5, 5.5, and 7 days, respectively.

For participants with IU, for whom assessments and calculated averages for W1 through W4 were available, changes over time with respect to the RESQ-7, the number of events, and the number of tablets taken were additionally analyzed by repeated measurement ANOVA and two-sided post-test according to Dunnett comparison of all weekly assessments (D7, D14, D21, D28) versus baseline. Single missing items for the RESQ-7 were implied using the mean score of the non-missing item scores of the individual. In case of missing data for whole domains at single assessments, the last observation carried forward principle was applied. If a domain was missing for D0, the last observation carried backward principle from D7 was applied.

Global assessments, impact of Refluthin use on overall rating of QoL, and safety parameters were analyzed descriptively.

## Results

### Study participants

**Fig. 1:**
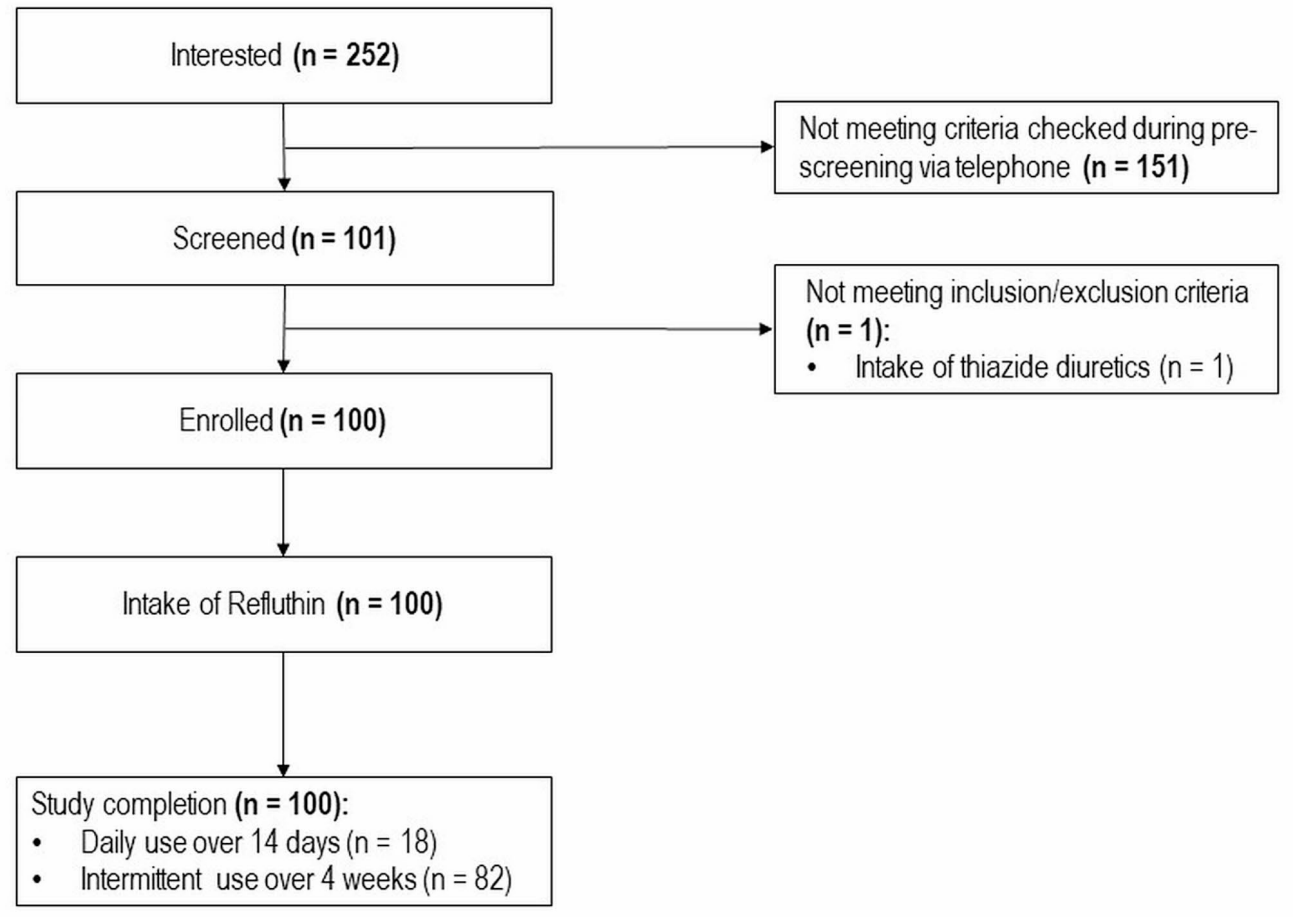
Study flow chart.

Out of the 252 pre-screened subjects, 101 could be invited for the screening visit (Fig. 1). One subject was not eligible due to intake of thiazide diuretics, all others were enrolled and included for ITT/FAS and safety set. A total of 18 participants completed the study earlier after taking Refluthin daily over 14 days. All other participants used Refluthin intermittently over the 4 weeks period. 53% of participants opted for fruit flavor, 47% for mint flavor.

Demographic and baseline characteristics are shown in Table 1. A higher frequency of heartburn events prior to the study was reported for participants with DU compared to IU (61% vs. 15% of participants with heartburn events at least once a day). Most participants reported symptoms like heartburn, regurgitation of air and/or acid stomach contents moving upwards or acid and/or bitter taste in the mouth. Fatty meals, very large meals in the evening, and consumption of sweets were the most frequently mentioned heartburn triggers.

**Table 1:** Demographics and baseline characteristics (N = 100; ITT/FAS population; absolute and relative numbers or means and standard deviation; IU, intermittent use; DU, daily use).

Demographics	All	IU	DU
	(n = 100)	(n = 82)	(n = 18)
Sex			
Male	47 (47%)	39 (48%)	8 (44%)
Female	53 (53%)	43 (52%)	10 (56%)
Age, years	52.9 ± 15.3	51.3 ± 15.3	60.3 ± 13.3
Body mass index, kg/m^2^	27.3 ± 4.9	27.0 ± 4.6	28.8 ± 5.8
Smoking habits			
Non-smoker	87 (87%)	71 (87%)	16 (89%)
< 10 cigarettes/d	6 (6%)	6 (7%)	0 (0%)
≥ 10 cigarettes/d	7 (7%)	5 (6%)	2 (11%)
Alcohol consumption			
No alcohol	16 (16%)	13 (16%)	3 (17%)
< once/week	42 (42%)	34 (41%)	8 (44%)
< a bottle of beer or 1/4L of wine/d	33 (33%)	27 (33%)	6 (33%)
> a bottle of beer or 1/4L of wine/d	9 (9%)	8 (10%)	1 (6%)
Coffee consumption			
No coffee	11 (11%)	11 (13%)	0 (0%)
< 1 cup of coffee/d	13 (13%)	10 (12%)	3 (17%)
1–2 cups of coffee/d	52 (52%)	43 (52%)	9 (50%)
≥ 3 cups of coffee/d	24 (24%)	18 (22%)	6 (33%)
Baseline characteristics			
Frequency of heartburn events prior to study			
1–3 times/week	48 (48%)	47 (57%)	1 (6%)
4–6 times/week	29 (29%)	23 (28%)	6 (33%)
Once daily	6 (6%)	3 (4%)	3 (17%)
Several times daily	17 (17%)	9 (11%)	8 (44%)
Short-GER questionnaire sum score, points	10.3 ± 3.6	10.0 ± 3.7	11.9 ± 3.0
Most frequently reported heartburn symptoms*			
Heartburn	84 (84%)	71 (87%)	13 (72%)
Regurgitation of air or acid stomach contents moving upwards	81 (81%)	65 (79%)	16 (89%)
Acid or bitter taste in the mouth	71 (71%)	56 (68%)	15 (83%)
Burning feeling behind the breastbone	62 (62%)	52 (63%)	10 (56%)
Main heartburn triggers*			
Fatty meals	67 (67%)	57 (70%)	10 (56%)
Spicy meals	46 (46%)	37 (45%)	9 (50%)
Very large meals in the evening	57 (57%)	46 (56%)	11 (61%)
Consumption of sweets	55 (55%)	43 (52%)	12 (67%)
Alcohol consumption	54 (54%)	47 (57%)	7 (39%)
Coffee consumption	46 (46%)	36 (44%)	10 (56%)
Lying down	52 (52%)	43 (52%)	9 (50%)
Quality of life			
Excellent	6 (6%)	6 (7%)	0 (0%)
Very good	34 (34%)	28 (34%)	6 (33%)
Good	41 (41%)	33 (40%)	8 (44%)
Fair	16 (16%)	12 (15%)	4 (22%)
Poor	3 (3%)	3 (4%)	0 (0%)

*Mentioned by ≥ 50% of participants in the total study group or at least one subgroup (multiple answers per participant possible).

Prior to the study, about two third of participants had used either prescription or OTC products for treatment of heartburn and alleviation of symptoms (acc. to Short-GER questionnaire; data not shown). A total of 19 participants reported former PPI use, but the last intake was longer than 4 weeks before start of the study. Medical history revealed metabolism and nutrition disorders like thyroid diseases or hyperlipidemias to be the most frequent diseases, followed by immune system disorders like allergies (data not shown). Therefore, concomitant therapy, besides vitamins, consisted mainly of thyroid therapies and lipid modifying or blood pressure modifying agents.

Characterization of study participants via GER questionnaire revealed that, prior to study inclusion, most of them complained about a sensation of pain, pressure, or burning starting in the stomach and spreading cranially in the thorax at least twice a week or once a month to once a week. Daily occurrence was reported by 10% of participants. A burning sensation deep in the throat was reported to appear rarely by 25%, once a month to once a week by 23%, at least twice a week by 26%, and daily by 10% of participants. A bitter, salty, or sour taste in the mouth mostly appeared once a month to once a week in 26% and at least twice a week in 30% of participants, while 15% of participants experienced this symptom daily. Moreover, 40% and 15% of participants perceived a feeling of regurgitation caused by food eaten some time ago at least twice per week or daily, respectively. A total of 52% of participants reported to have woken up at night feeling heartburn, coughing, or a choking throat in a frequency of once a month to once a week or even more often. The mean Short-GER questionnaire sum score of the participants (Table 1) corresponded to a medium intensity score.

### Number of events and days with an event

During the study, 1,464 heartburn events occurred, resulting in an average of 14.6 [95% CI: 13.1–16.2] events per study participant. Participants with daily Refluthin use reported twice as much heartburn events compared to those with IU (24.9 [20.5–29.3] vs. 12.4 [11.1–13.7] events). On average, heartburn events occurred on 11.2 [10.4–12.0] days; participants with DU had more days with heartburn events than those with IU (mean frequency 14.7 vs. 10.4 days).

In patients with IU, the number of documented heartburn events per week decreased over time (4.0 events in week 1 to 2.8 events in week 4). In patients with DU, an increase in the number of heartburn events was observed 11.0 events in week 1 to 13.9 events in week 2). For participants for whom the assessments of W1 to W4 were available (i.e., participants with IU), the decrease was significant in W2 (*p* < 0.01), W3 (*p* < 0.001), and W4 (*p* < 0.001) compared to W1.

### Number of tablets and intake per event

During the study, participants took an average of 15.4 [95% CI: 13.8–17.0] Refluthin tablets. Overall, 25 participants used more than one tablet of Refluthin for a single event at least once. None of the participants used more than two tablets of Refluthin for a single event or more than four tablets/day. In both subgroups, the median number of tablets taken per heartburn event was 1. The median number of tablets taken per week was 2 to 4 for IU compared to 11 and 14 for DU.

In participants with IU, the average weekly intake of Refluthin decreased from 4.2 [3.6–4.7] (W1) to 3.1 [2.5–3.6] tablets (W4). Participants with DU took an average of 13.9 [11.1–16.8] tablets in W2 compared to 11.3 [9.4–13.2] in W1.

### Intensity of heartburn symptoms

Between W1 and W4, the mean intensity of heartburn symptoms before intake of Refluthin decreased from 5.1 [95% CI: 4.8–5.5] to 4.5 [4.1–5.0] points in the total study population. In participants with IU, there was a decline from 5.2 [4.8–5.5] to 4.5 [4.1–5.0] points for this time period. Participants with DU showed a mean decrease in intensity of heartburn symptoms prior to Refluthin intake from 4.9 [4.1–5.8] (W1) to 4.5 [3.5–5.5] points (W2). After Refluthin intake, the intensity of heartburn symptoms was reduced by an average of 71.0% [66.8–75.3%] in the total study population. In participants with IU, the symptom relief was more pronounced compared to those with DU (73.0% [68.8–77.2%] vs. 62.1% [48.2–76.1%]).

The intensity of heartburn symptoms which the participants perceived after having achieved a tolerable level also slightly decreased over time, with a mean intensity of 1.4 [1.2–1.7%] (W1) compared to 1.2 [0.9–1.6%] points (W4). Participants with IU showed comparable results. In the DU group, no change was seen between the two weeks observed.

The significant reduction of heartburn intensity after Refluthin use was confirmed for the whole study period as well as within the single weeks of evaluation, i.e., intensity before intake versus intensity after tolerable level (*p* < 0.0001, IU, for all comparisons). Moreover, 82% of participants responded to Refluthin intake in terms of a reduction in pain intensity of ≥ 50% in ≥ 50% of reported events over the whole observation period.

### Time to onset of first relief

**Fig. 2:**
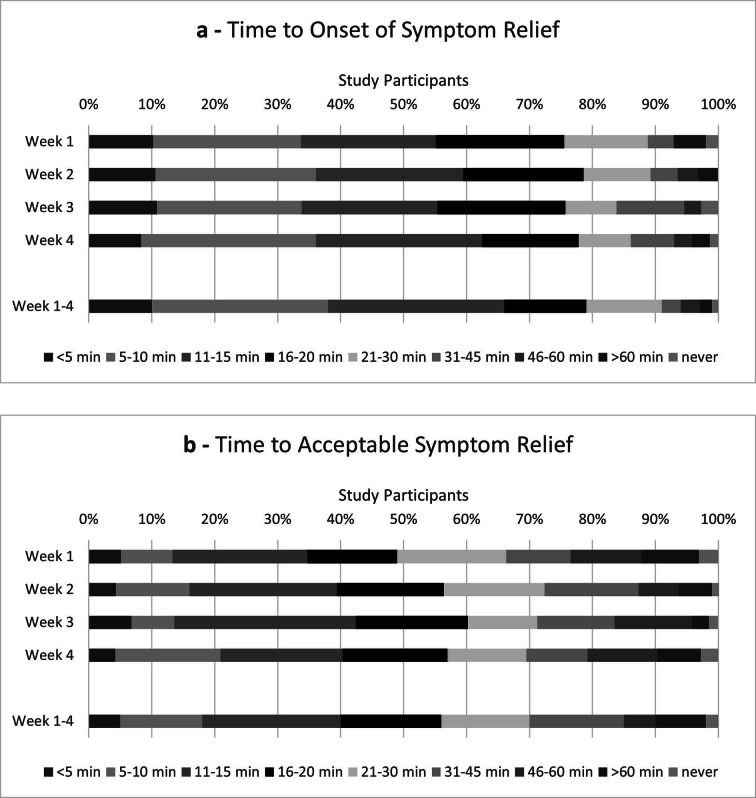
(**a**) Time to Onset of First Relief as rated by the participants (relative numbers of participants and individually calculated median categories; ITT/FAS; total study population; week 1–4: n = 100, week 1: n = 98, week 2: n = 94, week 3: n = 74, week 4: n = 72). (**b**) Time to Acceptable Symptom Relief as rated by the participants (relative numbers of participants and individually calculated median categories; ITT/FAS; total study population; week 1–4: n = 100, week 1: n = 98, week 2: n = 94, week 3: n = 73, week 4: n = 72).

In most cases, the time to onset of first symptom relief lay within 5–15 min during the 4-week observation period (Fig. 2A). This was also true for each individual week. The median value calculated for the whole observation as well as per week corresponded to a median duration of 11–15 min until onset of first relief. For the IU and DU subgroups, comparable timelines were reported, except for participants with DU in W2, for whom a median time to onset of 5–10 min could be seen. Within the cut-off category of “first relief in ≤ 10 min in ≥ 50% of reported events over the whole observation period”, already 39% of participants were classified as responders and within the time category “ ≤ 20 min”, a ratio of 81% was seen.

### Time to acceptable symptom relief

During the complete observation phase of 4 weeks, the time to an acceptable level of symptom relief was most frequently (in 22% of participants) reported as 11–15 min (Fig. 2B). This was also confirmed for the individual weeks. The median value calculated for the whole observation period corresponded to a median duration of 16–20 min until acceptable symptom relief. For the individual weeks, median durations of 21–30 min (W1) and 16–20 min (W2, W3, and W4) were reported. For participants with IU and DU, comparable timelines could be seen (data not shown). Within the cut-off category of “acceptable symptom relief in ≤ 20 min in ≥ 50% of reported events over the whole observation period”, already 56% of participants were classified as responders and within the time category “ ≤ 30 min”, a ratio of 72% was seen.

### Duration of effect

**Fig. 3:**
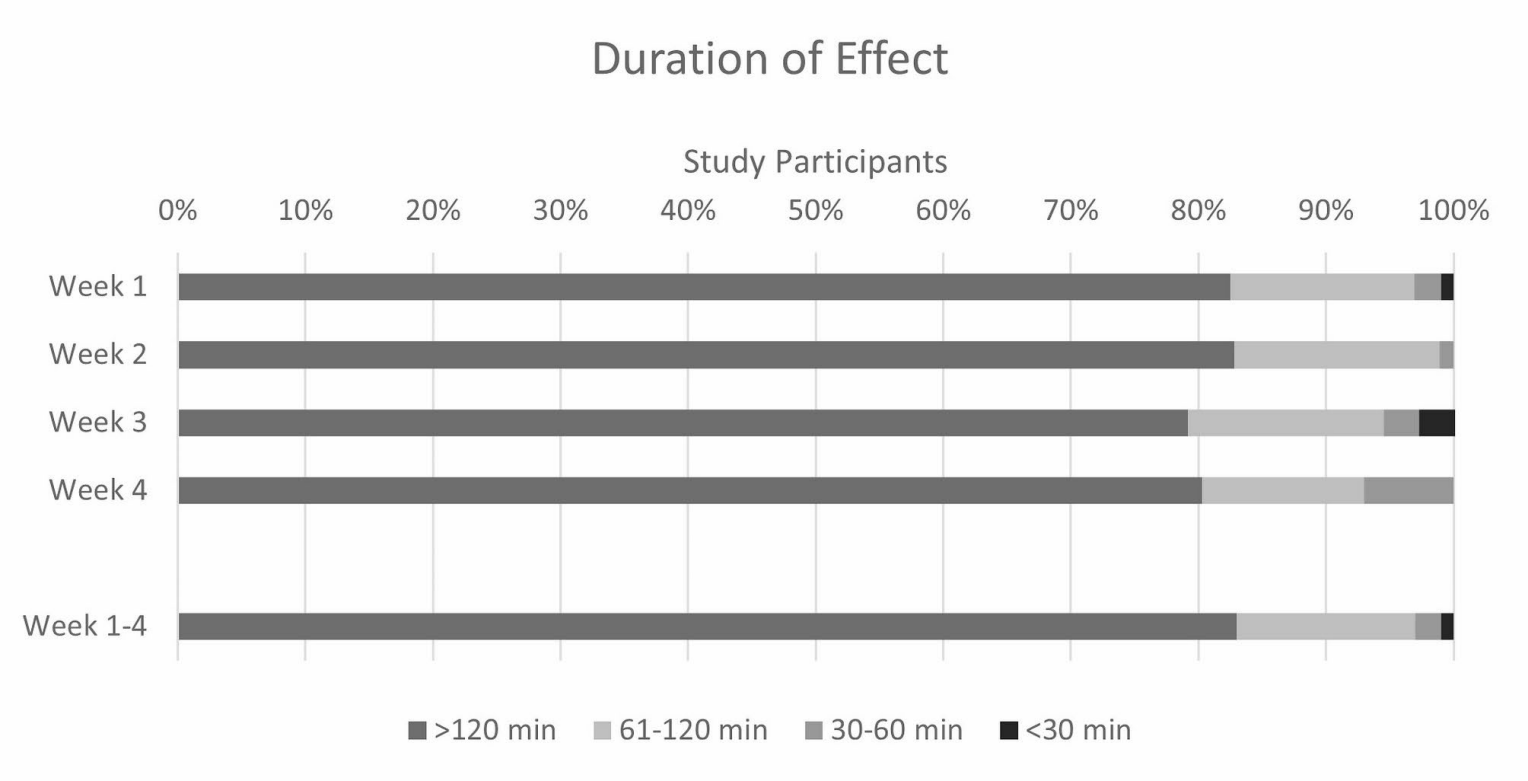
Duration of effect as rated by the participants (relative numbers of participants and individually calculated median categories; ITT/FAS; total study population; week 1–4: n = 100, week 1: n = 97, week 2: n = 93, week 3: n = 72, week 4: n = 71).

During the 4-week observation period, the duration of effect was most frequently reported as more than 120 min (83% of participants; Fig. 3). Comparable results were also found for the individual weeks. The median time category of the duration of effect was “more than 120 min” for the total study population and for both subgroups. Within the cut-off category of “duration of effect > 120 min in ≥ 50% of reported events over the whole observation period”, 81% of participants were classified as responders.

### Symptom frequency and intensity (RESQ-7)

Table 2 summarizes RESQ-7 OSDS results for frequency and intensity in the total group as well as in the IU and DU subgroups. On D0, mean values of symptom frequency per week and intensity of symptoms were higher in participants with DU compared to those with IU. Over the 4 weeks’ course of the study, a consistent improvement in the RESQ-7 OSDS for both frequency and intensity could be seen for the total group and the IU subgroup. In the subgroup with DU, symptom frequency did not change, while symptom intensity decreased slightly.

**Table 2:** RESQ-7 (means [95% CI]; RESQ-7—Mean overall symptoms domain scores for frequency and intensity rated by participants at days 0, 7, 14, 21, and 28 (maximum frequency: 7 days/week, maximum intensity: 5 points).

RESQ-7 overall symptoms domain	Group	Day 0	Day 7	Day 14	Day 21	Day 28
Frequency	TP	1.9 [1.6; 2.1]	1.7 [1.5; 1.9]	1.5 [1.3; 1.8]	1.2 [0.9; 1.4]	1.0 [0.8; 1.2]
	IU	1.7 [1.4; 1.9]	1.5 [1.3; 1.7]	1.3 [1.1; 1.5]	1.1 [0.9; 1.3]	1.0 [0.8; 1.2]
	DU	2.7 [2.2; 3.3]	2.8 [2.2; 3.3]	2.5 [1.7; 3.2]	2.7*	NA
Intensity	TP	1.9 [1.7; 2.0]	1.6 [1.4; 1.7]	1.4 [1.3; 1.6]	1.2 [1.0; 1.3]	1.0 [0.8; 1.2]
	IU	1.8 [1.6; 2.0]	1.5 [1.3; 1.6]	1.4 [1.2; 1.5]	1.2 [1.0; 1.3]	1.0 [0.8; 1.2]
	DU	2.2 [1.8; 2.6]	1.9 [1.6; 2.3]	1.7 [1.3; 2.1]	2.0*	NA

Higher scores indicate more intense or more frequent symptoms, respectively (ITT/FAS; total population [TP, n = 100] and subgroups for intermittent use [IU, n = 82] and daily use [DU, n = 18]).

*One participant of the subgroup with daily use ended use of Refluthin at the end of week 2 but returned the filled-out RESQ-7 for day 21, which was the day the final visit was scheduled.

The reduction over time was significant for both frequency and intensity (*p* < 0.0001 each; IU) with significant differences versus baseline at all time points (frequency—D7: *p* < 0.05; D14, D21, D28: *p* < 0.001 each; intensity—*p* < 0.001 at all 4 time points).

### Global assessments and willingness to recommend

Overall, 85% of participants (IU 87%, DU 77%) assessed the performance of Refluthin as very good or good (Table 3). The reasons given by participants for their assessment were mostly “quick effect” and “long lasting”.

**Table 3:** Global assessment of performance, global assessment of satisfaction, and willingness to recommend (study end; n = 100; ITT population; absolute and relative numbers) as well as global assessment of tolerability (study end; n = 100; safety population; absolute and relative numbers).

Global assessment	All	IU	DU
	(n = 100)	(n = 82)	(n = 18)
Performance			
Very good	47 (47%)	39 (48%)	8 (44%)
Good	38 (38%)	32 (39%)	6 (33%)
Moderate	12 (12%)	8 (10%)	4 (22%)
Poor	3 (3%)	3 (4%)	0 (0%)
No effect	0 (0%)	0 (0%)	0 (0%)
Satisfaction			
Very satisfied	59 (59%)	50 (61%)	9 (50%)
Satisfied	34 (34%)	26 (32%)	8 (44%)
Indifferent/no decision	5 (5%)	4 (5%)	1 (6%)
Unsatisfied	2 (2%)	2 (2%)	0 (0%)
Willingness to recommend to family member/friend			
Would strongly recommend	59 (59%)	49 (60%)	10 (56%)
Would recommend	35 (35%)	28 (34%)	7 (39%)
Indifferent/no decision	5 (5%)	4 (5%)	1 (6%)
Would not recommend	1 (1%)	1 (1%)	0 (0%)
Tolerability by participant			
Very good	91 (91%)	75 (91%)	16 (89%)
Good	6 (6%)	4 (5%)	2 (11%)
Moderate	2 (2%)	2 (2%)	0 (0%)
Poor	1 (1%)	1 (1%)	0 (0%)
Tolerability by investigator			
Very good	94 (94%)	77 (94%)	17 (94%)
Good	3 (3%)	2 (2%)	1 (6%)
Moderate	3 (3%)	3 (4%)	0 (0%)
Poor	0 (0%)	0 (0%)	0 (0%)

**Fig. 4:**
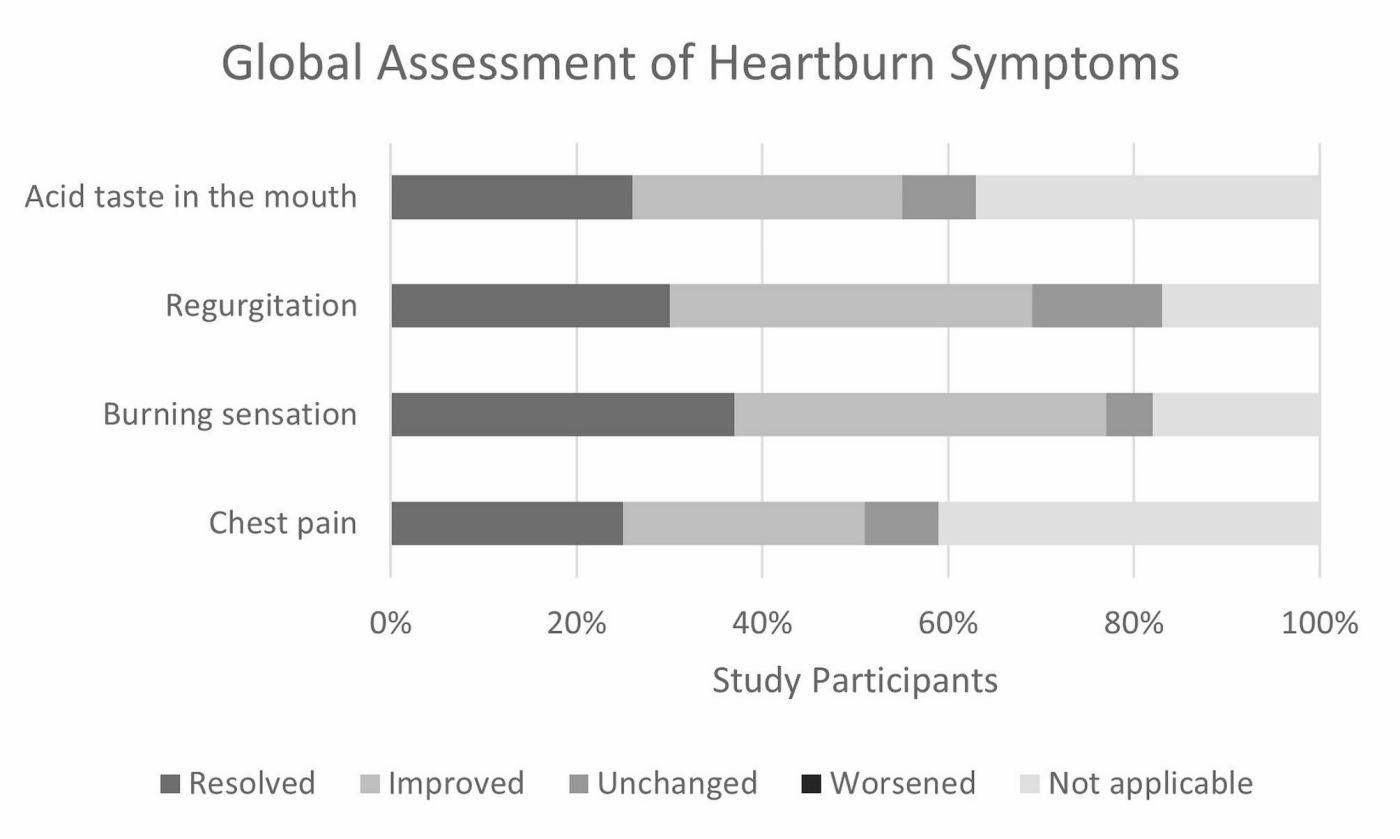
Global assessment of treatment effects based on single heartburn symptoms (relative numbers of participants; ITT/FAS; total study population; n = 100).

In the global assessment of treatment effects based on single heartburn symptoms (Fig. 4), no participant reported a worsening of symptoms and only a minority declared these symptoms as unchanged. Improved symptoms were reported by most participants and some symptoms were even judged as resolved.

According to the global assessment of satisfaction (Table 3), 93% of participants (IU 93%, DU 94%) were very satisfied or satisfied with the performance of Refluthin. Reasons given were mostly “high performance”, “fast relief”, and “good taste”.

**Fig. 5:**
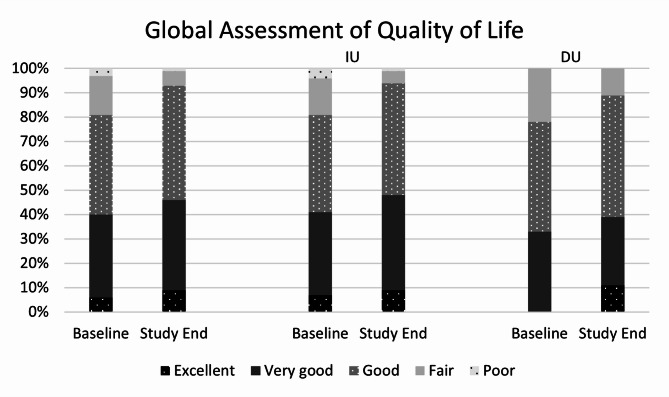
Global assessment of quality of life by participants at baseline and at study end (IU = intermittent use, DU = daily use; relative numbers of participants; ITT/FAS; total study population; n = 100).

Compared to study start (Table 1), more participants rated their QoL as excellent (i.e., increase from 6 to 9%), very good (34% to 37%), or good (41% to 47%) at the end of the study (Fig. 5). This resulted in 93% of participants with a good to excellent QoL rating at study end, with improvements compared to baseline in both subgroups. When asked whether the regular use of Refluthin had an impact on their QoL, 55% (IU 57%, DU 44%) answered yes (all other participants answered “no” to this question).

In the global assessment of application and user-friendliness, 99% of participants rated Refluthin as easy to use. Only one (1%) participant found the product not easy to use, resulting from a misinterpretation of the IFU, and consistently waited for one hour after food consumption prior to using Refluthin. This participant also rated Refluthin as not easy to integrate into daily routine, while a total of 97% of participants rated Refluthin use as easy to integrate (indifferent/no decision: 2%). The IFU was assessed as easy to understand by 92% of participants, 8% were indifferent or undecided.

Moreover, most participants would strongly recommend or recommend Refluthin to a family member or friend (Table 3).

### Safety and tolerability

Overall, 64 AEs were reported for a total of 42 participant. Respiratory, thoracic, and mediastinal disorders (e.g., common colds, sore throats, corona infection) were the most frequently mentioned category (n = 19), followed by nervous system disorders (e.g., headaches) (n = 17) and gastrointestinal disorders (n = 11). A total of 4 AEs reported by 3 participants were judged as ADEs but not as related to the study procedure. The causality was rated as probable in 3 (nausea, n = 2; abdominal discomfort on day of Refluthin intake, n = 1) and as possible in 1 ADE (abdominal discomfort). No device deficiencies were reported.

Global assessment of tolerability as rated by participants and investigator were also favorable (Table 3).

## Discussion

Heartburn and acid related gastric discomfort are common gastrointestinal symptoms, affecting millions of people around the world. In this context, many patients decide to try over-the-counter (OTC) products such as antacids first before seeking medical attention^10,31^. OTC products available for heartburn and acid related gastric discomfort should therefore be safe in use while providing a rapid and long lasting symptomatic relief. In the present study, both requirements are clearly met by Refluthin.

Study participants profited from the use of Refluthin by a rapid and long lasting relief in heartburn and acid related gastric discomfort. More than 80% of participants responded to Refluthin intake, i.e., they perceived a symptom relief within 20 min or faster in more than half of the individual applications, and just as many participants reported long lasting effects of more than 2 h. These results are in line with those of a recently published quantitative survey, in which more than 90% of users reported that Refluthin had helped them relieve their discomfort and just as many reported a soothing of the esophagus and stomach, respectively, and rated the speed to onset of effect after Refluthin intake positively^32^. It can therefore be concluded that a duration of up to 20 min until symptom relief as seen in the present study is an acceptable waiting period for the patients. The global satisfaction of participants observed in the formerly published survey and in the present study, respectively, also supports this assumption (positive ratings in 90% and 93% of participants, respectively). The same applies to the willingness to recommend Refluthin to others, which was correspondingly and comparably high at study end in both the formerly published survey and the present investigation (93% and 94%, respectively).

CaCO_3_ and MgCO_3_ increase pH quickly^13,33^, while the *Opuntia ficus-indica* extract component alone does not exhibit any acid-buffering effect^10,23,24^. Moreover, for Refluthin, it was shown that its Opuntia component does not negatively influence the acid-buffering effect of the antacid contained^23,24^. In stirred artificial gastric juice, the addition of a single Refluthin tablet suspended in 5 mL artificial saliva led to a rapid increase to pH 5.3 within 3 min, indicating a rapid dissolution of the suspension and neutralization of acid by the carbonate constituents^23^. The rapid relief observed in our study is therefore likely to be attributed to the antacid component of Refluthin. In an open, randomized, placebo-controlled trial in healthy volunteers, two tablets of a slightly higher dosed antacid (CaCO_3_, 680 mg, and MgCO_3_, 80 mg) achieved the target pH of > 3.0 during the first 30 min after intake, with a significant effect compared to placebo seen 10 min after intake^13^. This corresponds well with the results of our study, which reveal a duration of 11–15 min until onset of first relief, a duration of up to 20 min until acceptable symptom relief, and 39% of participants being responders within the first 10 min after intake.

However, the effect of the antacid tablets observed in the above-mentioned placebo-controlled open trial and in a similar investigation was reported to be only apparent within the first 30 min^13,33^. In fact, the effectiveness of antacids in general with respect to providing a more sustained relief of heartburn has not been adequately demonstrated yet^12^. The participants of our study, on the contrary, also profited by a long lasting relief of more than 2 h. The long lasting relief seen is therefore unlikely to be explained by the effects reported for antacids in the two studies mentioned above^13,33^. An explanation could rather lie in the mucoprotective properties reported for the *Opuntia ficus-indica* extract contained in Refluthin^22–24^. The protection of epithelial cell integrity observed non-clinically could, according to the authors’ interpretation^23,24^, underlie the described gastroprotective effect of *Opuntia* polysaccharides via the formation of a physical protective film^34^ and both components of Refluthin could contribute to the alleviation of heartburn via complementary mechanisms^23,24^. In fact, a protective activity of *Opuntia ficus-indica* extracts was already shown in animal models of experimentally induced gastritis and moreover demonstrated to be mediated by the high-molecular polysaccharide-containing mucilage fraction^15,17–19^. As revealed by in vitro studies, Opuntia polysaccharides form large colloidal aggregates in solution^35^ and bind to Caco-2 cells^34^, which suggests their ability to form viscous, physically acting protective films on the surface of gastrointestinal epithelial cells. There are no in vivo data available for adhesion time of Opuntia polysaccharides to esophageal or gastric mucosa yet, but evaluation of adhesive time of natural polysaccharides on Caco-2 cell line showed that 34% of reduction of lectin binding (i.e., indirect measurement of the binding of polysaccharides to the cell) was still measurable after washing experiments in Franz cells after 30 min^34^.

The clinical relevance of these non-clinically shown protective activities can be derived from double-blind, randomized-controlled studies investigating different products containing a polysaccharide-enriched Opuntia extract and showing efficacy in patients suffering from GERD and gastrointestinal discomfort, respectively^20,21^. As reported by Malfa and colleagues^21^, a 4-week treatment with a standardized extract of *Opuntia ficus-indica* L. cladodes and *Olea europaea* L. leaves in adults with gastrointestinal discomfort led to a reduction in heartburn and reflux, while there was no change in these symptoms in placebo-treated patients. After 8 weeks of treatment with a combination of sodium alginate/bicarbonate and *Opuntia ficus-indica* and *Olea europaea*, the mean number of heartburn and acid regurgitation episodes per week decreased by only one in placebo-treated GERD patients, while there were significant reductions of 5 to 6 episodes in patients treated with the combination product^20^. The effects observed with *Opuntia ficus-indica* extracts thus appear to be longer lasting than the short-term effects that could be shown for antacids alone^12,13,33^.

Based on the available data summarized above, it seems plausible that the mechanism of the observed beneficial effects of the *Opuntia ficus-indica* extract contained in Refluthin is a formation of highly viscous films on mucosal cells, which could act as a physical protective barrier, reduce access of noxious stimuli to the epithelial surface, and therefore may lead to long-time relief after use: In addition to the beneficial effects of Refluthin use seen in the present study after each individual intake, the symptom frequency and intensity reported by the participants decreased consistently during the course of the study, which also contrasts with the only temporary neutralization of gastric acidity and associated short-term improvement of symptoms as is described for antacids^13^. This is also in line with results from a non-clinical study reported recently^36^, in which a concentration-dependent protective activity of the *Opuntia ficus-indica* extract against bile acid-mediated esophageal cell irritation could be shown, compatible with the concept of formation of a physically acting barrier. The long-term effects observed in the present study also suggest that the highly concentrated *Opuntia ficus-indica* extract contained in Refluthin soothes and protects the irritated mucous membrane, leading to an acceleration of the re-epithelization of mucosal damage, if present. Although the present study was conducted without a placebo arm, it can nevertheless be stated that the results differ distinctly from those that can be expected from a natural course of the disease as shown by placebo results from former controlled clinical studies^20,21^. A benefit of mucosal protection in the relief of heartburn, epigastric pain/burning, or non-erosive reflux disease was already shown for other mucosal protective agents by randomized, controlled, double-blind, double-dummy, multicenter trials^37,38^. Corazziari and colleagues^37^ demonstrated a 2-week treatment with Poliprotect, i.e. a polysaccharide fraction derived from Aloe vera, Malva sylvestris, and Althea officinalis, along with minerals limestone and nahcolite, to be non-inferior to a standard dose of omeprazole in patients with heartburn and epigastric burning without erosive esophagitis or gastroduodenal lesions. Symptom relief was comparable, and the effect of Poliprotect persisted even after switching to on-demand use. Similarly, Savarino and colleagues^38^ could show that a hyaluronic acid-chondroitin sulphate based bioadhesive formulation (Esoxx) in addition to PPI resulted in significantly greater symptom relief than PPI monotherapy after two weeks of treatment.

For participants’ self-assessment of symptoms on a weekly basis, the RESQ-7^26^ was applied. The RESQ-7 covers different symptom domains (heartburn, regurgitation, burping as well as cough including hoarseness and difficulty of swallowing) from which a total score for intensity or frequency of symptoms can be generated. This score can be expressed in the form of average days per week and symptom and intensity categories, respectively. According to baseline results, each symptom perceived by the participants averagely occurred on nearly 2 days/week and was of mild intensity. RESQ-7 baseline data also showed that, as expected, the DU group suffered from a higher symptom burden than the IU group. The baseline differences seen for participants with DU and IU by means of the RESQ-7 are also reflected by further baseline data. Nearly two third of the DU group reported heartburn events to occur once or several times daily prior to the study, while this was the case in only about one of 7 participants of the IU group. However, no participant of the DU group reported poor QoL at baseline.

The baseline data discussed illustrate the suitability of the RESQ-7 for symptom assessment also in the present research framework and the plausibility of the study results obtained. Analysis of participants with IU, who could use Refluthin over the entire 4-week period, comprised the majority of the participants and showed gradual reductions in symptom intensity and frequency which reached statistical significance compared to baseline. Actually, participants with IU profited from Refluthin already after 14 days of use and, at D28, score reductions of almost 50% were seen, resulting in a mean frequency of 1 day/week and symptom as well as a “very mild” symptom intensity. In contrast, RESQ-7 values in the group of participants with DU, who could not use Refluthin over a full 4 weeks, did not improve comparably pronounced. While the reduction in intensity after 14 days was even slightly greater than in the IU group, the reduction in frequency was not quite as distinct. Due to the improvements seen in the IU group for both frequency and intensity already after 14 days, it cannot be assumed that any lack of improvement observed in the RESQ-7 is due to the shorter observation phase. At first glance, one might therefore conclude that Refluthin could not help users comparably well in the DU group due to their more severe symptoms. However, it should be kept in mind that the DU group was comparatively small. Moreover, participants in the DU group turned out to be comparably satisfied according to the global assessments performed at the end of the study. One possible reason for the favorable global assessments seen may therefore be that patients were satisfied with symptom control provided by Refluthin in such a way that they were happy to perceive about the same frequency of symptoms as usual, with less intensity, while they had to be less consistent in avoiding heartburn-triggers. However, to answer this question, further studies also including assessments of consumption habits over the whole study period would be necessary. In this context, it should also be noted that daily users did not finish use of Refluthin after 14 days of consecutive use because they considered it ineffective. Instead, they had to complete their final visit, as daily use beyond 14 days is not recommended in the IFU. This is to prevent milk-alkali syndrome (Burnett’s syndrome), which is traditionally associated with excessive intake of milk and absorbable alkalis^39^.

The significant reduction of heartburn events as well as the reduction in intensity prior to intake of Refluthin over time were in line with event diary results. It can thus be stated that data from both the prospective and retrospective assessment tools applied confirm the study findings and, therefore, underline the internal validity of the study results. This is also true for the improvement seen in QoL both in the total group and in the two subgroups. In fact, 19% of participants reported their QoL to be only fair or even poor at study start. This proportion decreased to only 7% at study end, and it should be noted that, at this timepoint, the rating “poor” was given only once. Moreover, the majority of participants explicitly affirmed an improvement in QoL at study end. These results suggest that patients can benefit from Refluthin regarding their QoL, irrespective of whether the latter is slightly or severely impaired at the beginning of use. Moreover, by examining normal Refluthin use according to the IFU, our study made it possible to take a look at the different needs of patients, clearly indicating that they adapted the dosage to their needs. This in in line with previous study results for PPI showing that on-demand treatment can be effective in controlling symptoms of nonerosive reflux disease and that many patients find it convenient and acceptable^40^.

Overall, the present study results on Refluthin reflect the dual mechanism of action reported^23,24^, i.e., reflux of acidic stomach content being quickly neutralized by mineral buffers^13,23^, leading to a fast relief of acute symptoms such as heartburn and acid regurgitation, and in addition, protection of the affected mucosa by a soothing film, which is likely to be mediated by the plant-based protective substances from the highly concentrated *Opuntia ficus-indica* extract^23,24,34^. The principal mode of action of Refluthin is thus primarily neither a metabolic, pharmacological, or immunological interaction with the human body, but of a physico-chemical nature. Refluthin users benefit in form of a rapid and long lasting relief and an improvement in symptom frequency and intensity, accompanied by a corresponding improvement in QoL.

In this respect and with regard to the primary goals for self-treating heartburn as suggested by the World Gastroenterology Organization^41^, i.e., to become symptom-free and restore one’s QoL, the results of our study are to be considered clinically relevant. Our results are also of clinical significance on the basis of the requirements of the German S2k guideline^8^, as data show distinguished effects on symptoms with which the patient is satisfied in the sense of an adequate symptom control. The study outcomes underline the beneficial effects of Refluthin for the patients affected by heartburn and acid related gastric discomfort and suggest Refluthin as a good option for use in the relief of their symptoms, neutralizing reflux of acidic stomach content quickly and leading to a rapid relief of symptoms such as heartburn and acid regurgitation.

Study participants had not been asked for how long they had been suffering from heartburn/GERD prior to the study and they decided individually when to take Refluthin and therefore on the symptoms and their severity that prompted them to do so. In the context of a study conducted after the product has been placed on the over-the-counter market, this is a suitable setting in order to be able to map usage by patients on their own responsibility according to the IFU.

By both the participants and the investigator, the global tolerability of Refluthin was reported to be very good or good in most participants. No serious AEs occurred during the study and no device deficiencies were reported. The good tolerability seen is also in line with safety results from former clinical studies investigating products with *Opuntia ficus-indica* extract^20,21^. It is also worth emphasizing the fact that most study participants confirmed the easy and user-friendly application, which was additionally underlined by the fact that the study participants found the application of Refluthin easy to be integrated in daily routine.

When discussing the results of our study, some potential limitations should be considered. It should be kept in mind that this was not a hypothesis-testing, randomized clinical trial. The study was designed to collect and evaluate clinical data on the safety and performance of Refluthin under conditions of normal use and, therefore, no placebo control group was included. All results have to be interpreted accordingly. By meta-analysis of randomized, placebo-controlled, double-blind clinical trials investigating proton-pump inhibitors and H2-receptor antagonists (H2RA), it has been shown that placebo response rates are substantial among GERD clinical trials, yet lower than that observed in other functional and inflammatory disorders of the gastrointestinal tract^42^ and that placebo response in GERD appears to be related to the class of acid suppression treatment studied. Nevertheless, the high number of reported events and the consistent improvement throughout the present study clearly support the reliability of our data. It should also be noted that the observed reduction in intensity of heartburn symptoms was over 60% in the total study group and in both subgroups. This is distinctively more than a 50% improvement in heartburn intensity, which is defined in the literature as a measure of a successful response to therapy^43–45^. Moreover, due to the non-confirmative design, there was no formal primary outcome variable, and most endpoints were based on self-assessment, relying on the report by the participants, and therefore inherently subjective. An indication of internal validity of the results, however, is given from the fact that a concurrent improvement could be seen regardless of whether the data were collected via prospective or retrospective assessment tools, as for example in relation to the reduction in heartburn events, the reduction in intensity prior to intake of the products, and the results derived from the event diary. Furthermore, the observed effects align well with non-clinical and clinical data from the literature as discussed above.

The study moreover included the use of the RESQ-7^26^. In a first step, this tool was useful for sample size calculation, as no applicable statements on clinical relevance in our patient group could be found from the literature. Therefore, data and assumptions from earlier reported trials in GERD patients^28,29^, which implied the Reflux Disease Questionnaire (RDQ) on which the development of the RESQ-7 was based, were taken into account. We also expected this standardized tool to be of additional value for the assessment of GER symptoms in the present study population although it was primarily intended to assess symptoms in GERD patients only partially responsive to PPI therapy. In this context, it should be kept in mind that, due to the non-confirmative study design and the focus on whether patients perceived benefit from sufficient symptom control in accordance with the German S2k guideline^27^, we were independent of the availability of a minimal clinical important difference for the tool to be used in the patient population we investigated. As shown by our results, the RESQ-7 total scores for intensity and frequency are indeed in good agreement with the course of symptoms as shown by the other parameters applied.

## Conclusions

The present study results demonstrate a safe use of and a distinct, rapid, and long lasting symptom relief after intake of Refluthin. The significant reductions in both frequency and intensity of heartburn events seen over the duration of use indicate sustained effects of this novel symptomatic treatment option under treatment. Thus, Refluthin can be safely used by subjects who suffer from heartburn, acid regurgitation, and/or acid related gastric discomfort.

## Data Availability

The datasets presented in this article are not readily available because raw data cannot be shared both due to ethical reasons and to data protection laws. To the extent permitted by law, the study data required for validation purposes have been disclosed on corresponding databases. Reasonable requests to access the datasets should be directed to the corresponding author.

## Abbreviations

ADEs: Adverse device effects
AEs: Adverse events
CaCO_3_: Calcium carbonate
D: Day
DRKS: German clinical trials register
DU: Daily use
FAS: Full analysis set
GER: Gastro-esophageal reflux
GERD: Gastro-esophageal reflux disease
IFU: Instructions for use
ISO: International Organisation for Standardization
ITT: Intention-to-treat
IU: Intermittent use
MgCO_3_: Magnesium carbonate
OSDS: Overall symptoms domain scores
OTC: Over-the-counter
PMCF: Post-market clinical follow-up
PPI: Proton pump inhibitors
QoL: Quality of life
RDQ: Reflux disease questionnaire
RESQ-7: Reflux symptom questionnaire 7-day recall
W: Week
